# 2003 Mixed meal effects of neprilysin inhibition

**DOI:** 10.1017/cts.2018.171

**Published:** 2018-11-21

**Authors:** Jessica R. Wilson, Daniel Rader, Raymond Townsend, Michael Rickels

**Affiliations:** 1 University of Pennsylvania School of Medicine; 2 Department of Medicine, Division of Nephrology and Hypertension, University of Pennsylvania; 3 Department of Medicine, Division of Endocrinology, University of Pennsylvania

## Abstract

OBJECTIVES/SPECIFIC AIMS: Test the hypothesis that neprilysin inhibition with sacubitril/valsartan will increase endogenous intact GLP-1 after a mixed meal compared with valsartan. METHODS/STUDY POPULATION: Adults 18–80 years with pre-diabetes or type 2 diabetes and elevated blood pressure. RESULTS/ANTICIPATED RESULTS: We anticipate higher intact GLP-1 area under the cure after the meal when subjects receive sacubitril/valsartan compared with valsartan. DISCUSSION/SIGNIFICANCE OF IMPACT: Neprilysin inhibition may be a target for anti-diabetes therapy by decreasing degradation of GLP-1.

